# *Amomum subulatum*: A treasure trove of anti-cancer compounds targeting TP53 protein using *in vitro* and *in silico* techniques

**DOI:** 10.3389/fchem.2023.1174363

**Published:** 2023-04-26

**Authors:** Sadaqat Ali, Asifa Noreen, Adeem Qamar, Imran Zafar, Quratul Ain, Hiba-Allah Nafidi, Yousef A. Bin Jardan, Mohammed Bourhia, Summya Rashid, Rohit Sharma

**Affiliations:** ^1^ Medical Department, DHQ Hospital Bhawalnagr, Punjab, Pakistan; ^2^ Department of Chemistry, Rippha International University, Faisalabad, Pakistan; ^3^ Department of Pathology, Sahiwal Medical College Sahiwal, Punjab, Pakistan; ^4^ Department of Bioinformatics and Computational Biology, Virtual University of Pakistan, Punjab, Pakistan; ^5^ Department of Chemistry, Government College Women University, Faisalabad, Pakistan; ^6^ Department of Food Science, Faculty of Agricultural and Food Sciences, Laval University, Quebec City, QC, Canada; ^7^ Department of Pharmaceutics, College of Pharmacy, King Saud University, Riyadh, Saudi Arabia; ^8^ Laboratory of Chemistry and Biochemistry, Faculty of Medicine and Pharmacy, Ibn Zohr University, Laayoune, Morocco; ^9^ Department of Rasa Shastra and Bhaishajya Kalpana, Faculty of Ayurveda, Institute of Medical Sciences, Banaras Hindu University, Varanasi, Uttar Pradesh, India

**Keywords:** *Amomum subulatum*, TP53, cancer, anti-cancer compounds, pharmacophore modeling, high-throughput techniques, bioinformatics

## Abstract

Cancer is a primary global health concern, and researchers seek innovative approaches to combat the disease. Clinical bioinformatics and high-throughput proteomics technologies provide powerful tools to explore cancer biology. Medicinal plants are considered effective therapeutic agents, and computer-aided drug design (CAAD) is used to identify novel drug candidates from plant extracts. The tumour suppressor protein TP53 is an attractive target for drug development, given its crucial role in cancer pathogenesis. This study used a dried extract of *Amomum subulatum* seeds to identify phytocompounds targeting TP53 in cancer. We apply qualitative tests to determine its phytochemicals (Alkaloid, Tannin, Saponin, Phlobatinin, and Cardic glycoside), and found that alkaloid composed of 9.4% ± 0.04% and Saponin 1.9% ± 0.05% crude chemical constituent. In the results of DPPH Analysis *Amomum subulatum* Seeds founded antioxidant activity, and then we verified via observing methanol extract (79.82%), BHT (81.73%), and n-hexane extract (51.31%) found to be positive. For Inhibition of oxidation, we observe BHT is 90.25%, and Methanol (83.42%) has the most significant proportion of linoleic acid oxidation suppression. We used diverse bioinformatics approaches to evaluate the effect of *A. subulatum* seeds and their natural components on TP53. Compound-1 had the best pharmacophore match value (53.92), with others ranging from 50.75 to 53.92. Our docking result shows the top three natural compounds had the highest binding energies (−11.10 to −10.3 kcal/mol). The highest binding energies (−10.9 to −9.2 kcal/mol) compound bonded to significant sections in the target protein’s active domains with TP53. Based on virtual screening, we select top phytocompounds for targets which highly fit based on pharmacophore score and observe these compounds exhibited potent antioxidant activity and inhibited cancer cell inflammation in the TP53 pathway. Molecular Dynamics (MD) simulations indicated that the ligand was bound to the protein with some significant conformational changes in the protein structure. This study provides novel insights into the development of innovative drugs for the treatment of cancer disorders.

## 1 Introduction

Cancer is a multifaceted ailment that has the potential to impact any anatomical structure, and it is the result of a combination of genotypic, environmental, and lifestyle determinants (Pandya et al., 2021; Mazhar et al., 2023). Although cancer may manifest in any individual, specific predisposing factors, such as advanced age, familial history, and exposure to carcinogenic substances, can augment the likelihood of developing the disease. Cancerous cells are atypical cells that multiply and proliferate in an unbridled fashion (Hossain et al., 2022). They can generate neoplasms, infiltrate adjacent tissues, and disseminate to other body regions via a phenomenon known as metastasis. Cancerous cells can disrupt the normal functioning of organs and tissues in the body, resulting in various symptoms and complications. Managing cancer is challenging because it isn’t a single disease but a group of illnesses with different causes, such as evidence from clinical presentations and treatment options (Lu et al., 2020). The treatment of cancers varies depending on their type and stage. Cutaneous carcinoma and haematological malignanciesrequire special treatments. Research in the field of cancer is ongoing, with new therapies and curative methods being developed continuously. The most promising areas of cancer research currently include immunotherapy, which uses the immune system to target and destroy cancer cells, and personalized medicine, which tailors treatment to the unique genomic and molecular characteristics of an individual’s cancer. These innovative approaches can potentially transform cancer treatment, leading to more effective and precise therapies that may improve outcomes and increase survival rates (Kaminski et al., 2020). Prevention is equally critical in combatting the ubiquitous impact of cancer, and this includes efforts to reduce exposure to known carcinogens, such as tobacco smoke and UV radiation, as well as promoting healthy lifestyles, such as regular exercise and a balanced diet (Balwan and Kour, 2021; Klein et al., 2022). In-depth, cancer is a complex and challenging disease. Still, with ongoing research, prevention efforts, and advances in treatment provides a hope for improving outcomes and reducing the impact of this disease on individuals and communities worldwide.

The p53 gene is responsible for encoding a vital transcription factor protein called p53 (Sharma et al., 2023), plays a crucial role in regulating essential cellular mechanisms such as DNA repair, cell cycle arrest, and programmed cell death (apoptosis). Upon sensing signals of DNA damage or similar stressful stimuli, the p53 protein can galvanize or inhibit the expression of sundry downstream genes, predicated on the prevailing cellular ambience. The p53 protein interacts with a vast spectrum of alternative proteins to form a labyrinthine network of interplays, which help to govern its activity and fuse its reaction to distinct stress signals (Chen L et al., 2020). These interactions and networks are complex and context-dependent, with a range of downstream targets and regulators that can influence its activity and function. Some essential proteins and networks associated with p53 include MDM2, ATM/ATR, p21, BAX, and NF-kB (Karimian et al., 2019; Samavarchi Tehrani et al., 2019; Marei et al., 2021). Anomalies in the TP53 gene have the potential to compromise the efficacy of the p53 protein, thereby inciting uncontrolled cellular multiplication and a heightened susceptibility to cancer. However, therapeutic techniques that target the p53 pathway are being pioneered to ameliorate curative alternatives for cancer patients. These comprise minuscule molecule medications that can rejuvenate or stabilize the p53 protein, gene manipulation methodologies that introduce operative copies of the TP53 gene into neoplastic cells, and immunotherapeutic manoeuvres that utilize the immune system to attack cancer cells bearing TP53 gene aberrations. A comprehensive comprehension of the interplays and networks linked with p53 is indispensable for the evolution of precision therapies and the augmentation of clinical outcomes for patients who have cancer or related ailments (Otohinoyi et al., 2022).

Herbal medicine utilizes natural compounds that interact harmoniously with our bodies, while conventional medicine relies on synthesized and artificial molecules. Herbal medicineHerbal medicine practitioners stress the importance of combining various medicinal ingredients instead of isolating a single component (Xijun et al., 2016; Ali et al., 2018). Herbal medications are often preferred by individuals who cannot tolerate pharmaceuticals or experience adverse reactions to them. Natural medicine target the underlying cause of symptoms in certain conditions, whereas medications only alleviate the symptoms. Synthetic treatments are associated with a range of adverse effects, from minor to severe, and cause harm to the body’s internal organs and external features, including the skin, hair, and teeth. Previous research {Sharifi-Rad, 2020 #2712} has shown that herbal medicine may be slower in producing results, but the outcome is healthier when using natural compounds and treatments. Artificial processing of natural substances can result in toxicity concerns, and ensuring that the proper dosage is administered is essential to guarantee safety. However, if herbal medicine is used carefully, it may be possible to develop more effective and less toxic pharmaceuticals than those solely relying on refined medications (Saad et al., 2017).

Traditional medicine heavily depends on plants and their bioactive constituents to treat various ailments, including cancer (Matowa et al., 2020). An estimated two-thirds to three-quarters of the global population utilize herbal remedies for therapy, leading to an upsurge in interest in studying phytomedicines and their active biological properties. Phytochemicals are non-nutrient bioactive molecules found in plant-based foods like fruits and vegetables and have been associated with reduced risks of chronic diseases (Wu et al., 2021). Plants contain approximately 25,000 terpenoids, 12,000 alkaloids, 8,000 phenolics, and other compounds, providing a bountiful source of active molecules. Understanding how essential compounds like flavonoids, chlorogenic acids, alkaloids, carotenoids, minerals, and toxic substances affect health results can be learned from data on these compounds. Recent studies by (de Carvalho and Conte-Junior, 2021) indicate that the potential health advantages of phytochemicals found in fruits and vegetables could be more significant than previously assumed. Antioxidants, for example, can help mitigate oxidative stress caused by free radicals, which can contribute to the progression of chronic diseases. However, many phytochemicals remain unidentified, requiring identification and measurement before assessing their health risks. *Amomum subulatum* is a short-lived herbal plant widely used globally for its culinary and medicinal properties. Its health-promoting effects have been documented in Ayurveda and traditional Chinese medicine, and *in-vivo* and *in-vitro* studies have validated its anticancer potential.

The domain of cancer research has undergone a revolution through in-silico approaches, which present a cost-effective and time-efficient alternative to traditional experimental methods (Rahman et al., 2022). In-silico approaches involve describing and simulating biological processes, predicting drug-target interactions, and using computational tools and algorithms to develop new antitumor drugs. Researchers can explore vast chemical regions and identify potential cancer-targeting drugs by utilizing molecular docking, molecular dynamics models, and virtual screening methods. These methods also enable the prediction of medication metabolic and toxicity characteristics, thereby reducing the risk of failure during clinical studies. Moreover, in-silico methods facilitate the detection of genomic and epigenetic alterations in cancer cells, which can aid in developing more personalized treatments. These techniques have shown tremendous potential in identifying new targets and creating innovative cancer therapies. In organic plant molecules, bioinformatics plays a critical role in detecting and treating cancer (Tabrez et al., 2022). Using computational tools and databases, scientists can proficiently explore the chemical space of natural plant compounds and their interactions with cancerous targets. Bioinformatics approaches also allow the discovery of potential synergistic amalgamations of plant compounds that enhance efficacy and mitigate toxicity (Fokunang and Fokunang, 2022). By scrutinizing the gene expression profiles of cancerous cells, bioinformatics can help recognize biomarkers that predict the response to plant-based therapies (Ahmad et al., 2022; Satpathy, 2022). Additionally, bioinformatics approaches can expedite the repurposing of existing drugs for cancer treatment and the detection of new targets for drug development. All in all, bioinformatics offers a powerful platform for leveraging the therapeutic potential of natural plant compounds for cancer therapy.

The principal aim of our study is to explore the feasibility of employing novel phytocompounds originating from the *A. subulatum* seeds as anti-cancer drugs by targeting the TP53 receptor. To attain this objective, we implement computational techniques for structure prediction and validation, molecular docking, and simulation studies to scrutinize the interaction between the selected phytocompounds and TP53. The investigation includes cherry-picking a set of phytocompounds from the *A. subulatum plant* with the potential to combat cancer. It will then assess their three-dimensional structures and accuracy verification through computational methods. The molecular docking analyses are carried out to scrutinize the binding interactions between the chosen phytocompounds and TP53. The primary goal is to recognize new phytocompounds with high binding affinity and specificity towards TP53. The outcomes obtained from the molecular docking studies are further endorsed through simulation studies to ascertain that the binding interactions are steady and biologically significant. To evaluate the potential of phytocompounds as anti-cancer medications, their ADMET characteristics, including uptake, diffusion, metabolism, elimination, and toxicity, are closely examined. Based on the results of these studies, phytocompounds with optimal pharmacokinetic and toxicity profiles are identified. In addition, by investigating the interactions of these phytocompounds with the TP53 receptor and analyzing their ADMET characteristics, researchers aim to gain new insights into the potential of phytocompounds derived from *the A. subulatum* seeds as anti-cancer agents. The findings of this study could pave the way for developing more sophisticated and effective cancer treatments.

## 2 Materials and methods

### 2.1 Plant materials

The *A. subulatum* seeds were randomly gathered from a particular location of the agricultural lands in Faisalabad. They were washed and dried at room temperature to ensure an optimal outcome and prevent impurities. For powder extraction, 75 g of the seeds were pulverized, mixed with Methanol, and agitated on an orbital shaker for 7 mins using the maceration method. As per the method of the earlier researcher (Kumar et al., 2022), we used maceration permits the solvent to infiltrate the plant material and dissolve the targeted components. The aqueous filtrate, which consists of the desired compounds extracted from the plant material, was attained by sieving the mixture to eliminate unwanted particles or impurities. To precisely measure the number of active compounds in the *A. subulatum* seeds, the extracted material was converted into a solid form utilizing an evaporation technique, which included heating the methanol solution to evaporate the solvent and leaving behind the solid components of the *A. subulatum* seed extraction. The aim of obtaining a solid form of the extraction is to simplify weighing and quantifying, thus making it more convenient for analysis.

### 2.2 Phytochemical Screening

We performed Phytochemical Screening to determine the presence of particular chemical compounds in the *A. subulatum* seeds, utilizing traditional techniques by the approach employed by preceding investigators (Cabero Pérez, 2020). These constituents consist of tannins, flobatannins, saponins, steroids, and terpenoids, which are all important bioactive compounds frequently present in plants and can offervarious health advantages. To discern the presence of tannins, the pulverized, desiccated seeds of *A. subulatum* were subjected to boiling in water, and the resultant admixture was filtered. A minute quantity of 0.1% FeCl_3_ was then appended, and the manifestation of a brown-green tint denoted the existence of tannins. Tannins are a polyphenolic compounds recognized for their antioxidative and antimicrobial characteristics (Leite et al., 2021). To identify flobatonins, a 0.5 g specimen of the seeds was boiled with 1% aqueous HCl and the presence of flobatonins was indicated by the emergence of a crimson precipitate. Flobatonins are a type of tannin that is present in various plant species and are acknowledged for their astringent properties. To detect saponins, 2 g of the specimen was heated in distilled water to extract the compounds. After filtration, 5 mL of distilled water was added to 10 mL of the filtrate, and the mixture was vigorously shaken to produce stable foam. Olive oil droplets were added to the frothy mixture and promptly mixed to form an emulsion. Saponins are a glycoside compound with diverse pharmacological activities, including anti-inflammatory and anti-cancer effects (El Aziz et al., 2019). 0.5 g of the ethanol extract was added into test tube with 2 mL of H_2_SO_4_ and 2 mL of acetic anhydride to screen for steroids. The change in colour from violet to green indicated the presence of steroids, a type of lipid molecule with diverse physiological functions, including the regulation of metabolism, immune response, and development (Morales-Lázaro et al., 2019). For terpenoids analysis, 5 mL of each extract was delicately mixed with 2 mL of chloroform and 3 mL of concentrated sulfuric acid to generate a layer. The reddish-brown staining of the interface suggested that terpenoids are a large class of natural compounds known for their diverse biological activities, including anti-inflammatory, anti-cancer, and anti-microbial effects (Fan et al., 2023).

### 2.3 DPPH analyses

DPPH radical scavenging is a commonly employed procedure for gauging the antioxidative efficacy of natural substances, including plant-based extracts as per earlier researchers (Amarowicz et al., 2004). DPPH is a stable radical that exhibits a unique absorption peak at 517 nm and can be rendered neutral by antioxidants, leading to a decrement in absorption intensity. To evaluate the antioxidant potential of *A. subulatum* seeds, their ability to neutralize DPPH (2,2-diphenyl-1-picrylhydrazyl) radicals were investigated. To achieve this, a solution of DPPH in Methanol was mixed and resulting mixture was then incubated for 30 min, after which the absorbance was measured at a wavelength of 517 nm. The scavenging ability of the extract against DPPH radicals was inferred from the reduction in absorption of the reaction mixture, and the percentage of DPPH radical scavenging activity was calculated accordingly. In this investigation, the synthetic antioxidant BHT was utilized as a positive control to compare the antioxidative activity of *A. subulatum* seeds with a recognized antioxidant. A higher percentage of DPPH radical scavenging activity corresponded to a more tremendous antioxidative potential of the extract (Pyrzynska and Pękal, 2013).

### 2.4 Reducing power determination

To assess the plant specimen’s ability to reduce, a modified rendition of the technique, initially presented by Matanjun et al. (2008), was utilized. Various dissolvable extractions of *A. subulatum* seeds were concocted at convergences that fluctuated between 2.5 and 1.0 mg/mL. Sodium phosphate buffer (5.0 mL) and potassium ferricyanide (5.0 mL, 1.0% in 0.2 M, pH 6.6) were added to every extraction. The resultant mixture was then left to incubate at a temperature of 50°C for 20 min. After incubation, the mixture was centrifuged at 980 *g* and 5°C for 10 min. The resulting supernatant was then treated with 5 mL of 10% trichloroacetic acidvia applying second round of centrifugation. The top layer of the resultant solution (roughly 5 mL) was diluted with 5 mL of distilled water and 1.0 mL of 0.1% ferric chloride. The spectrophotometer was then used to measure the absorbance of the solution at a wavelength of 700 nm. This protocol was conducted in order to ascertain the plant sample’s reducing power Pasaribu et al. (2021).

### 2.5 Hemolytic activity

The hemolytic efficacy of *A. subulatum* seeds was assessed using a customized methodology as delineated by Pasaribu et al. (2021). To prevent coagulation, 3 mL of human blood was collected into a heparinized tube and centrifuged at 850 *g* for 5 min. The resulting supernatant was discarded, and the red blood cells (RBCs) were washed three times with 5 mL of chilled sterile isotonic phosphate-buffered saline (PBS) solution at pH 7.4. The RBCs were then suspended in 20 mL of chilled PBS and counted using a hemacytometer. The RBC count was adjusted to 7.068 × 108 cells per mL to prepare for the assay. Plant extracts (20 µL) were instilled into 2 mL Eppendorf tubes, succeeded by adding diluted blood cell suspension (180 µL). The samples were incubated for 35 min at 37°C, followed by placing the tubes on ice for 5 min and centrifuging for 5 min at 1,310 g. After centrifugation, the supernatant (100 µL) was withdrawn from each tube and diluted with chilled PBS (900 µL). The mixture from each Eppendorf (200 µL) supernatant was then added to 96-well plates. For each test, 0.1% Triton X-100 was utilized as a positive control and PBS as a negative control. For hemolysis %, the absorbance was measured at 576 nm via appyingformula: % hemolysis = (sample absorbance/control absorbance) × 100. Tomaintain consistency during the assay, all Eppendorf tubes were kept on ice.

### 2.6 Structure prediction

The amino acid sequence of TP53, having accession number (P04637), was obtained from the UniProt database (https://www.uniprot.org/) in the FASTA format. Then we performed a BLASTp query against the Protein DataBank (https://www.rcsb.org/search) to locate fitting templates (6IU7, 6IUA, 6MY0, 5Z78, 5ZCJ, 6I3V, 7LIN, and 7LIO) for the desired protein. Utilizing SWISS model (https://swissmodel.expasy.org/) and ITASSER (https://zhanggroup.org/I-TASSER/) server, multiple 3D structures were prognosticated and validated. To appraise the quality of the envisaged 3D structures, numerous online validation tools, including ERRAT (https://saves.mbi.ucla.edu/), Verify3D, and Rampage (http://www.scfbio-iitd.res.in/software/proteomics/protsav.jsp), were employed. Chimaera was afterwards tooptimize the 3D structures of the selected compounds intermingled with TP53. While conducting the simulation studies, the protein secondary structure elements (PSSE) were thoroughly scrutinized and determined. These techniques facilitated the foreknowledge and validation of TP53’s 3D structures and the revelation of undiscovered phytocompounds possessing potential anti-cancer properties.

### 2.7 Docking analysis

For in-silico analysis, we utilized a reservoir of autochthonous compounds obtained from the Asinex database (https://www.asinex.com/). We select phytocompounds based on their pharmacophore fit score using AutoDock Vina and employed LigandScout for pharmacophore modelling (Wolber and Langer, 2005; Heider et al., 2022). We utilized AutoDock Vina for targeted molecular docking and meticulously examined and visualized the docking investigations employing UCSF Chimera v1.12 and Discovery Studio. We identified 2D structures from databases like PubChem (https://pubchem.ncbi.nlm.nih.gov/) to create plant-based molecules and employed ChemDraw and Chimera. We performed 100 docking runs for each docking experiment and added polar hydrogen atoms to all targeted proteins. We chose TP53 as the grid size for docking investigations. To ensure drug-likeness, we rigorously examined Lipinski’s Rule of Five (RO5) as per the method of earlier researcher Chen X et al. (2020), by utilizing the online mcule server (https://mcule.com/) and computed the drug characteristics of all specified compounds. To evaluate the ADMET properties (Absorption, Distribution, Metabolism, Excretion, and Toxicity), we utilized the AdmetSAR tool (http://lmmd.ecust.edu.cn/admetsar2) to scrutinize the bioavailability of the selected compounds.

### 2.8 Molecular dynamic simulation

Molecular Dynamic (MD) simulations have investigated the interplay between the protein and ligand (Salo-Ahen et al., 2020). The preeminent docked complex was handpicked, and various modules from the Schrodinger suite were utilized to execute the MD simulations (Páll et al., 2020). An NPT ensemble was implemented to prepare a rudimentary simulation milieu for the docked systems, held at a temperature of 300 K for a duration of 100 nanoseconds (ns) as per the method of earlier researcher Prunotto (2020). In protein-ligand docking, the docked conformers underwent root mean square deviation (RMSD) and root mean square fluctuation (RMSF) plot analysis to evaluate the stability and fluctuations of the protein-ligand complex during the MD simulation. The RMSD analysis quantifies the structural aberration between the docked complex and the MD trajectory, whereas the RMSF analysis gauges the residue-wise fluctuation of the complex throughout the simulation as per the methods of earlier researchers (Rather et al., 2020; Tabti et al., 2023). The MD simulation outcomes were assessed utilizing various visualization tools, including VMD and Chimera, to acquire perspicacity into the protein-ligand interactions and recognize prospective binding sites and vital residues implicated in the interaction.

## 3 Results and discussion

### 3.1 Phytochemical Screening of *Amomum subulatum* seeds

This study scrutinised the existence of phytochemicals in *A. subulatum* seeds by conducting various qualitative tests to assess their plausible therapeutic advantages. Qualitative phytochemical analysis was executed, which divulged the presence of tannins, saponin, steroids, and cardiac glycosides in the seeds, whereas phlorotannins, terpenoids, and alkaloids were not detected. Phytochemicals such as saponin and tannins are renowned for their inherent antibiotic properties and are frequently employed to combat pathogenic strains. Alkaloids exhibit confirmed antioxidant activities and have been noted as efficacious therapeutic agents in ethnomedicine. Tannin has been implemented as an active compound in pharmaceuticals and beverages, primarily because of its antioxidant effects, as per the investigation by Saxena et al. (2013). Flavonol glycosides, among others, are potent inhibitors of lipid peroxidation. Steroids can scavenge free radicals and convert them into more stable molecules, arresting the chain reaction. Cardiac glycosides have been utilized to treat moderate to severe myocardial infarctions by inhibiting the Na+/K+ pump and increasing Ca++ concentrations. These phytochemicals offer myriad health benefits, such as anticancer properties, cholesterol-lowering effects, promotingng strong bones, and boosting the immune system (Felth et al., 2009). Saponins possess many activities, including anti-inflammatory, antifungal, hemolytic, fungistatic, molluscicidal, and foaming properties. The results of this study are consistent with previous studies (Wang et al., 2011; Abbas et al., 2015), suggesting that *A. subulatum* seeds are rich in phytochemical constituents that may be responsible for the antioxidant and anticancer activities of plant-based foods. The present study on *A. subulatum* has identified several therapeutically active components in the plant, as exhibited in Table 1. Alkaloids, tannins, saponins, and cardiac glycosides were detected in the plant, while steroids and terpenoids were absent.

**TABLE 1:** Qualitative analysis of the phytochemicals of the *Amomum subulatum*seeds extracts.

Plant	Alkaloid	Tannin	Saponin	Steroid	Phlobatinin	Terpenoid	Cardic glycoside
*Amomum subulatum*	+	+	+	—	+	—	+

In this study, the crude chemical constituents of *A. subulatum* seeds were quantitatively estimated, as shown in Table 2. At the same time, qualitative parameters were evaluated to distinguish between closely related plant species or varieties with similar pharmacological activities. The plant contained alkaloids, tannins, and saponins, which possess medicinal properties and are used in various antibiotics to treat common pathogenic strains. Previous research has also demonstrated the presence of alkaloids in leafy vegetables, such as the bitter leaf, which have been reported to alleviate headaches associated with hypertension.

**TABLE 2:** Quantitative estimation of the percentage crude chemical constituents in *Amomum subulatum* seeds extracts.

Chemical constituents of *Amomum subulatum*	Percentage (%) of crude chemical constituent
Alkaloids	9.4 ± 0.04
Saponin	1.9 ± 0.05

Alkaloids are a group of nitrogen-containing chemical substances that frequently have medicinal effects. Alkaloids have a wide range of molecular structures in numerous organisms. *Amomum subulatum* has an estimated alkaloid concentration of 9.40.04%, which is a substantial quantity. This shrub contains alkaloids like piperine, amomumine, and aphylline. Another family of chemical substances discovered in vegetation are saponins. They have a range of biological functions and are distinguished by their capacity to produce steady froths when agitated with water. Researchers have discovered that saponins have antifungal, antibacterial, and anti-inflammatory properties. According to reports, *A. subulatum* contains 1.90.05% saponin. Cardamonin, quercetin, and kaempferol are a few saponins that can be found in this plant. Further research is required to fully comprehend the molecular components’ precise impacts and possible applications in *A. subulatum*, which may have therapeutic qualities.

### 3.2 Percentage yield of plant extracts

The study found that the amount of plant compounds present varied between 2.72 and 3.97 mg/100 g of dried plant material. The methanol extract of *A. subulatum* seeds, which is known to be effective in extracting phytochemical components, yielded the highest amount of compounds. However, the choice of solvent can significantly impact the number of recovered substances since different solvents can extract distinct phytochemical components depending on their charges. Therefore, it is crucial to carefully consider the choice of the solvent when removing plant chemicals to ensure the best output and effectiveness of the extracted components. The percentage yield of plant extracts, which represents the number of separated chemicals produced from a specific volume of starting plant material, is anessential factor to consider in the extraction process. Since various solvents with different phases can extract distinct phytochemical components, the solvent option for plant extraction can substantially impact the number of recovered substances. Plant extract output in this situation differed according to the extraction fluid. For instance, the output for n-hexane was only 1.5, which isn’t very high. This is because n-hexane is a non-polar solvent that works well to remove non-polar substances like triglycerides and essential oils. However, removing polar substances like flavonoids and phenolics might not be as efficient.

**FIGURE 1:**
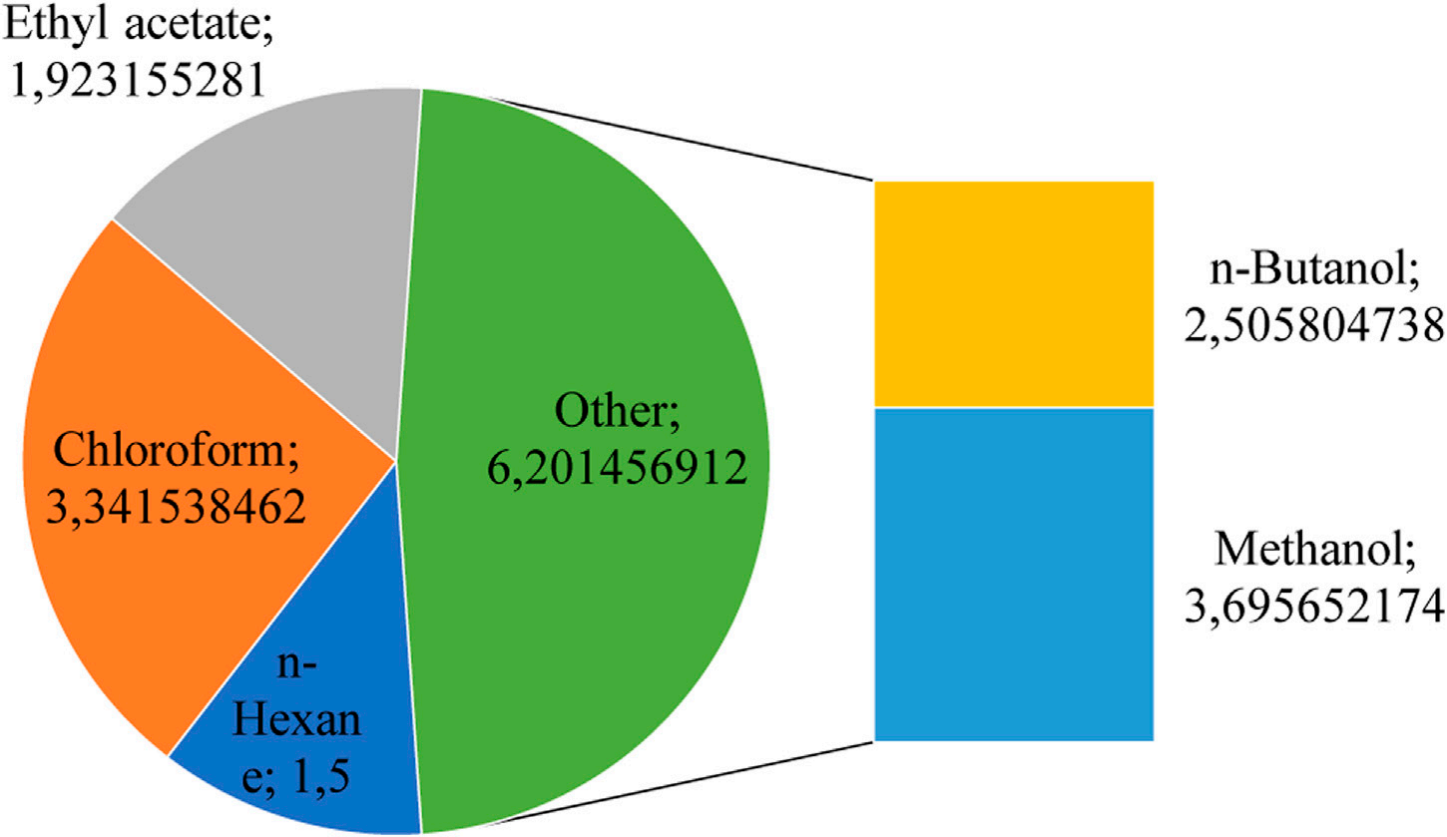
Percentage yield of *Amomum subulatum* seeds extracts.

On the other hand, the output of chloroform was 3.5, which is a significant value. A reasonably neutral fluid like chloroform can be used to remove a variety of chemicals from plant matter successfully. The use of chloroform should be avoided if feasible because it is a dangerous chemical. With yields of 2.5, acetone and butanol can remove plant chemicals with a tolerable efficiency level. While butanol is a less polar solvent that is effective in extracting non-polar substances like terpenoids and alkaloids, acetone is a polar solvent that removes polar substances like flavonoids and phenolics. According to Figure 1, Methanol had the most incredible output out of the 4. Various phytochemical components can be extracted from plant material using the neutral liquid Methanol. Methanol is poisonous and explosive, so it should be closely regulated and supervised.

#### 3.2.1 Total flavonoids content

Table 3 (A) presents the cumulative flavonoid matter (CFM) detected in arid essences of *A. subulatum* seeds through varying solvents employed for the extraction phase. CFM is expressed in catechin equivalencies (CE) per 100 g of arid core, where our results divulge that CFM values are contingent on the solvent selected for the extraction process. Our data indicate that the CFM for the n-hexane extract was found to be the least at 4.47 ± 0.05 mg/100 g dry extract, whereas the CFM for the methanol extract was the highest at 127.51 ± 0.76 mg/100 g dry extract. The chloroform, ethyl acetate, and n-butanol extracts exhibited CFM values of 32.17 ± 0.52, 17.48 ± 0.17, and 25.98 ± 0.50 mg/100 g dry extract, respectively. Our results signify that Methanol is the most proficient solvent for flavonoid extraction from *A. subulatum seeds*, whereas n-hexane is the most inefficient. Flavonoids are vital secondary metabolites in plants that augment plant colouration and exhibit diverse salutary biological activities, including anti-inflammatory, anti-allergic, and anti-cancer properties (Ekalu and Habila, 2020). Our findings suggest that the solvent preference for plant extraction can significantly influence the cumulative flavonoid content in the extract. Therefore, it is imperative to cautiously consider the solvent selection for each extraction to guarantee optimal efficiency and yield of the extracted flavonoids.

**TABLE 3:** 3 (A) explore the Total flavonoid contents, 3 (A) Total phenolic contents, 3 (B) Total phenolic contents, 3 (C) DPPH percentage scavenging assay, 3 (D) linoleic acid percentage inhibition and oxidation and 3 (E) explore the hemolytic activity in the percentage of hemolysis in extracts of *Amomum subulatum* seeds extracts.

Labels	3-A	3-B	3-C	3-D	3-E
Sample	Total Flavonoid Contents (CE Mg/100 G) Dry Extracts	Total Phenolic Contents (GAE Mg/100 G) In Dry Seeds Extracts	DPPH Percentage Scavenging	Linoleic Acid Percentage Inhibition and Oxidation	Percentage Of Hemolysis
n-Hexane	4.47 ± 0.05	23.25 ± 0.102	51.31 ± 0.38	23.68 ± 0.53	1.44 ± 0.019
Chloroform	32.17 ± 0.52	111.55 ± 0.136	60.02 ± 0.58	56.92 ± 0.61	3.11 ± 0.024
Ethyl acetate	17.48 ± 0.17	26.95 ± 0.056	72.41 ± 0.76	45.70 ± 0.32	4.19 ± 0.043
n-Butanol	25.98 ± 0.50	125.09 ± 0.101	68.96 ± 0.66	73.50 ± 0.53	6.10 ± 0.05
Methanol	127.51 ± 0.76	134.39 ± 0.589	79.82 ± 0.54	83.42 ± 0.87	8.50 ± 0.072
BHT/Titron X 100	—	—	81.73 ± 0.79	90.25 ± 0.90	99.64 ± 0.92

#### 3.2.2 Total phenolics content

Our research study explored the total phenolic content (TPC) of extracts obtained from *A. subulatum* seeds using different solvent mediums, primarily focusing on methanol extraction. TPC was quantified in milligrams of gallic acid equivalents (GAE) per 100 g of dry plant material. Findings showed that the TPC varied considerably across different solvent systems, with the highest TPC being recorded in the methanol extract (134.39 ± 0.589 mg GAE/100 g). In contrast, the n-hexane extract recorded the lowest TPC (23.25 ± 0.102 mg GAE/100 g) as elaborated in Table 3 Colum (B). Phenolic compounds are well-known for their remarkable antioxidant properties. The higher TPC content in the extracts significantly contributes to improved, reducing power and radical scavenging effect on DPPH radicals (Muzolf-Panek and Stuper-Szablewska, 2021). We used the Folin-Ciocalteu method to measure TPC, a sensitive, interference-free, and rapid method for quantifying the number of phenolics present in plant extracts. Phenolics are extensively found in plants and have been associated with diverse biological activities, including antioxidant activity (Karak, 2019). They act as reducing agents, hydrogen donors, and oxygen quenchers, thereby effectively decreasing oxidative stress. Our analysis concludes that the TPC of the extracts varied significantly across different solvent systems, with methanol extraction resulting in the highest TPC and phenolic content significantly contributing to their antioxidant activities.

### 3.3 DPPH analysis of *Amomum subulatum* seeds for antioxidant activity

The DPPH test is a popular technique for evaluating the antioxidant activity of natural substances. It considers a material’s capacity to bind free radicals and convert them to a rigid state. The more free radicals the material scavenges, the more antioxidant activity it exhibits. The DPPH% scavenging technique was used in this study to assess the antioxidant potential of different formulations of *A. subulatum* seeds. The results demonstrate variations in the antioxidant activity of *A. subulatum* seed products. The methanol extract (79.82%) and BHT (81.73%), a synthetic antioxidant used as a standard, had the highest percentage of scavenging activity. The least quantity of scavenging activity was demonstrated by the n-hexane extract (51.31%). These results suggest that the methanol seed extract of *A. subulatum* has considerable antioxidant activity.

The TPC and TFC assays reveal that phenolic and flavonoid compounds are what give the products their antioxidant properties. The preparation’s high content of total phenols may be responsible for their better results in terms of reducing power and radical cooling effect on DPPH radicals. The antioxidant qualities of phenolic compounds are due to their ability to give electrons, which allows them to combat free radicals and serve as reducing agents. Our findings of this study point out the significant antioxidant activity of *A. subulatum* seed products, with the methanol extract having the highest antioxidant capacity. These discoveries depend on developing organic antioxidants and using Amomum subulatum seeds in foods and medications.

The proportion of DPPH free radical scavenging activity of various solvent preparations of *A. subulatum seeds*, such as n-hexane, chloroform, ethyl acetate, n-butanol, Methanol, and BHT, is shown in Table 3 Column (C) as a synthetic antioxidant. The methanol extract had the most significant proportion of antioxidant action (79.82%), followed by BHT (81.73%). The reducing activity of the n-hexane extract was the lowest (51.31%). According to the study, the antioxidant potential of the compounds, especially phenolics, relies on their capacity to function as hydrogen-donating species. Their lowering ability may also influence the antioxidant activity of the compounds. The findings indicate that various liquid preparations of *A. subulatum seeds* had different antioxidant effects on DPPH (Rafique et al., 2020). The total phenol concentration, flavonoid content, radical scavenging activity, and % suppression of linoleic acid oxidation were all at their highest levels in the methanol extract. Due to their capacity to serve as a supply of antioxidant compounds, the results imply that the seeds of *A. subulatum* have strong antioxidant activity.

The results demonstrate that the DPPH test is an accurate technique for detecting phenolic and flavonoid components in natural goods and the antioxidant capabilities of mass preparations. In percentage words, the extract’s capacity to absorb free radicals was concentration-dependent, and this capacity increased with extract volume and hydroxylation level. The methanol extract had the highest scavenging activity, but it had slightly lower antioxidant activity than the synthetic antioxidant BHT. The study concludes that *A. subulatum* seeds have a variety of antioxidant impacts on DPPH, with the methanol extract having the strongest lowering action. It is advised to use the DPPH test as a valuable tool to assess the antioxidant capabilities of mass products. The study highlights the potential of *A. subulatum* seeds as an antioxidant chemical source (Pyrzynska and Pękal, 2013).

### 3.4 Inhibition of oxidation

The current study work’s findings imply that preparation from *A. subulatum* seeds has antioxidant qualities that can prevent oxidative damage, a phenomenon that can harm biological cells. Linoleic acid was used as a model system in the research to evaluate the degree to which compounds inhibited oxidation. According to the study’s findings, the various *A. subulatum* seed preparations inhibited oxidation to differing degrees. According to Table 3 Colum (D), methanol extract had the most significant proportion of oxidation suppression, suggesting the most potent antioxidant activity. This outcome might result from the methanol extract having a higher quantity of phytochemical components.

In contrast, n-hexane extract exhibited the lowest percentage of suppression, likely due to the non-polar solvent’s reduced phenolic and other phytochemical components content. It’s important to note that BHT, a manufactured antioxidant frequently employed in the food business, was used to compare the antioxidant activity of the compounds to that of BHT. According to the findings, only the methanol extract’s antioxidant activity was marginally lower than that of BHT’s, while that of all other extracts was noticeably lower. The findings imply that the extract from Amomum subulatum seeds has antioxidant qualities that can prevent lipid degradation. The of phytochemical components in the extract and the extraction liquid affect how much suppression occurs.

The proportion suppression of linoleic acid oxidation in various liquids is shown in Table 3 Colum (D), along with a positive control of BHT (butylated hydroxytoluene). A mixed fatty acid called linoleic acid is vulnerable to oxidation, which creates free radicals that can harm cells and tissues through oxidative stress. N-hexane, formaldehyde, ethyl acetate, n-butanol, and Methanol were among the solvents examined. Methanol (83.42%) has the most significant proportion of linoleic acid oxidation suppression, followed by n-butanol (73.50%), ethyl acetate (45.70%), chloroform (56.92%), and n-hexane (23.68%). The percentage reduction for the positive control, BHT, is 90.25%. These findings imply that the examined solvents can block linoleic acid oxidation to varying degrees, with Methanol and n-butanol exhibiting the greatest inhibiting action. The well-known antioxidant BHT is most suppressed, demonstrating its potent antioxidant activity. These findings may significantly impact the application of solvents and antioxidants in the culinary, medicinal, and skin care sectors.

### 3.5 Reducing power

**FIGURE 2:**
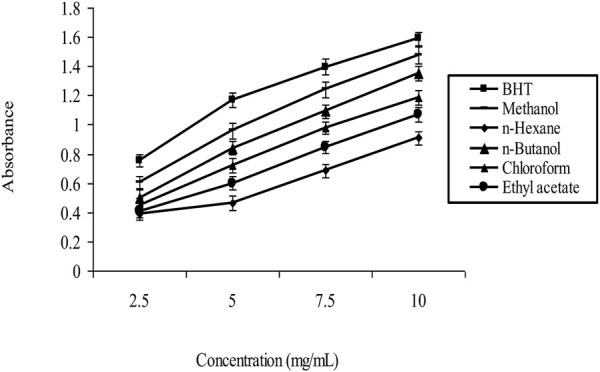
Reducing power by *Amomum subulatum*seedsextracts.

According to the study’s results, *A. subulatum* seeds’ natural antioxidant properties appear significant. They can be used to prevent the harm that free radicals and lipid breakdown cause to living cells. To evaluate how well the various formulas stopped lipid breakdown, linoleic acid was used as a model system. As shown in Figure 2, our findings revealed that the methanol extract had the largest% oxidation decrease, suggesting the most potent antioxidant activity. The greater quantity of phytochemical components in the methanol solution may be responsible for this outcome. Due to the low content of phenolic and other phytochemical components in this non-polar fluid, the n-hexane extract had the lowest proportion of oxidation suppression. These findings imply that the number of phytochemical components in the extract, which can impact the extract’s antioxidant activity, can be influenced by the pH of the liquid used during the extraction process.

It is also notable that BHT, a synthetic antioxidant frequently used in the food business, was used to measure the goods’ antioxidant activity. The findings revealed that all compositions had considerably reduced antioxidant activity than BHT, except the methanol extract, which had marginally lower antioxidant training than BHT. This contrast demonstrates the effectiveness of using natural antioxidants, such as those present in *A. subulatum*, instead of manufactured ones. The combined findings imply that the seeds combination of *A. subulatum* may be used as a natural supply of antioxidants to prevent lipid degradation and free radical harm. Additional investigation is required to determine the most efficient extraction technique and pinpoint the particular phytochemical components accountable for the purported antioxidant activity.

### 3.6 Hemolytic activity for cytotoxicity assay

According to our findings, *A. subulatum* seeds have naturally existing antioxidants that can stop lipid peroxidation, a process that can be harmful to living cells. When using linoleic acid as a model system to assess the ability of the different extracts to avoid oxidation, the study results showed that the methanol extract had the highest antioxidant activity. The greater concentration of phytochemical components in the methanol extract may cause the extract’s enhanced antioxidant activity, which is noted in Table 3 Colum 3 (E). The decreased antioxidant activity observed in the n-hexane extract is presumably due to the lower concentration of phenolic and other phytochemical components in this non-polar liquid. These findings suggest that the extraction solvent may affect the amount of phytochemical components in the extract and, as a result, on the extract’s antioxidant activity.

Comparing the antioxidant activity of the molecules to that of BHT, a synthesised antioxidant, provides a helpful insight into the potential use of natural antioxidants as an alternative to manufactured antioxidants. The potential that natural antioxidants could be used as effective alternatives to synthetic antioxidants is raised by the methanol extract’s slightly decreased antioxidant activity compared to BHT. However, the significantly reduced antioxidant activity discovered in the other formulations suggests that additional research is needed to determine the most effective extraction method and to identify the same phytochemical elements responsible for the reported antioxidant activity. According to our research findings, *A. subulatum* seeds contain natural antioxidants that can stop lipid degradation and protect cells from damage caused by free radicals. More research is necessary to fully understand the potential of these natural antioxidants and determine the most efficient extraction and application techniques.

### 3.7 Virtual screening analyses

**FIGURE 3:**
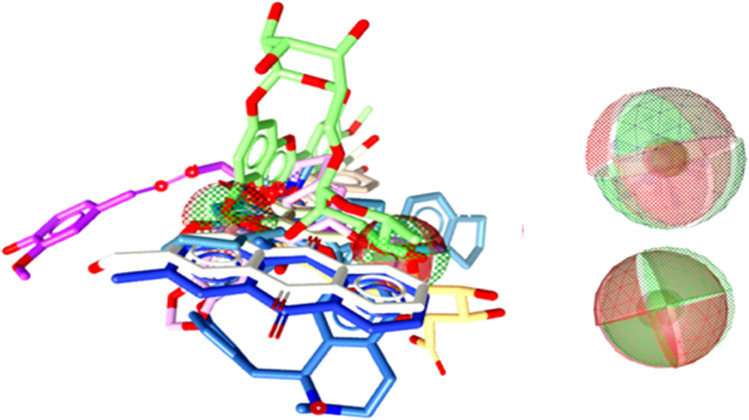
3D representation of pharmacophore modelling.

Computer-aided drug design (CADD) has emerged as a promising approach for discovering novel plant-based cancer treatments. Computational drug design involves using computational methods to identify potential drug candidates from libraries of natural plant compounds. Natural compounds with anticancer properties can be starting points for drug design studies. The TP53 protein, which plays a crucial role in cancer, is a key therapeutic target. In this study, homology-based modelling was used to create 3D structures of the TP53 protein based on its sequence and protein templates with high sequence identity and query coverage were selected, as shown in Figure 3. The top 10 lead hits from virtual screening analyses were subjected to pharmacophore modelling to identify the 3D arrangement of chemical features necessary for these compounds to interact with the TP53 protein. The resulting pharmacophore models could guide the design of new small molecule inhibitors of the TP53 protein for the treatment of cancer.

Pharmacophore refers to a 3D arrangement of chemical features necessary for a molecule to interact with a specific biological target. In the case of virtual screening of P53 protein, the top 10 lead hits are likely small molecules that have been predicted to bind to the protein based on their expected shape, electrostatic properties, and other characteristics. Based on previous studies of the P53 protein, it is known that the protein has several pockets and grooves on its surface that are important for interactions with other molecules. These pockets and grooves have specific shapes and electrostatic properties that can be used to guide the design of small molecule inhibitors. The pharmacophore matches findings for the chosen substances revealed encouraging outcomes, as shown in Table 4. Out of all the implications, Compound-1 had the best pharmacophore match value (53.92). In summary, the pharmacophore of the top 10 lead hits from virtual screening analyses of P53 protein likely includes features that allow them to interact with specific pockets and grooves on the protein’s surface.

**TABLE 4:** Pharmacophore of top 10 lead hits from virtual screening analyses and Calculated binding affinities of 10 lead hits docked compounds against P53.

Sr #	Top leads hit from virtual screening	Highest score of top 10 leads hits	Binding affinities (kcal/mol)
1	Compound-1	53.92	−11.10
2	Compound-2	51.05	−10.7
3	Compound-3	51.09	−10.3
4	Compound-4	51.07	−8.4
5	Compound-5	51.78	−6.9
6	Compound-6	50.75	−7.7
7	Compound-7	51.06	−7.6
8	Compound-8	51.07	−7.10
9	Compound-9	51.80	−7.6
10	Compound-10	50.75	−7.7

A pharmacophore is a molecular model that describes the spatial arrangement of the functional groups in a molecule that is responsible for its biological activity. It can identify small molecules that share similar structural features to known ligands or bind to a specific target. In this case, a pharmacophore model was generated for the top 50 lead hits identified in the virtual screening study, and the highest-scoring compounds among the top 10 were selected. The table lists with the highest score for each of the top 10 compounds identified in the virtual screening study. The score reflects the fit of the compounds to the pharmacophore model, with higher scores indicating a better fit. Based on the table, the top 10 lead hits have scores ranging from 50.75 to 53.92, with compound-1 having the highest score. These compounds were selected as the most promising hits based on their high scores and are thus expected to have a higher likelihood of binding to the target protein with high affinity. It is important to note that a high pharmacophore score does not guarantee that a compound will be an effective drug. Further studies, such as molecular docking and molecular dynamics simulations, are necessary to assess the binding and stability of the compounds to the target protein and evaluate their potential as drug candidates.

### 3.8 Molecular docking analyses

**FIGURE 4:**
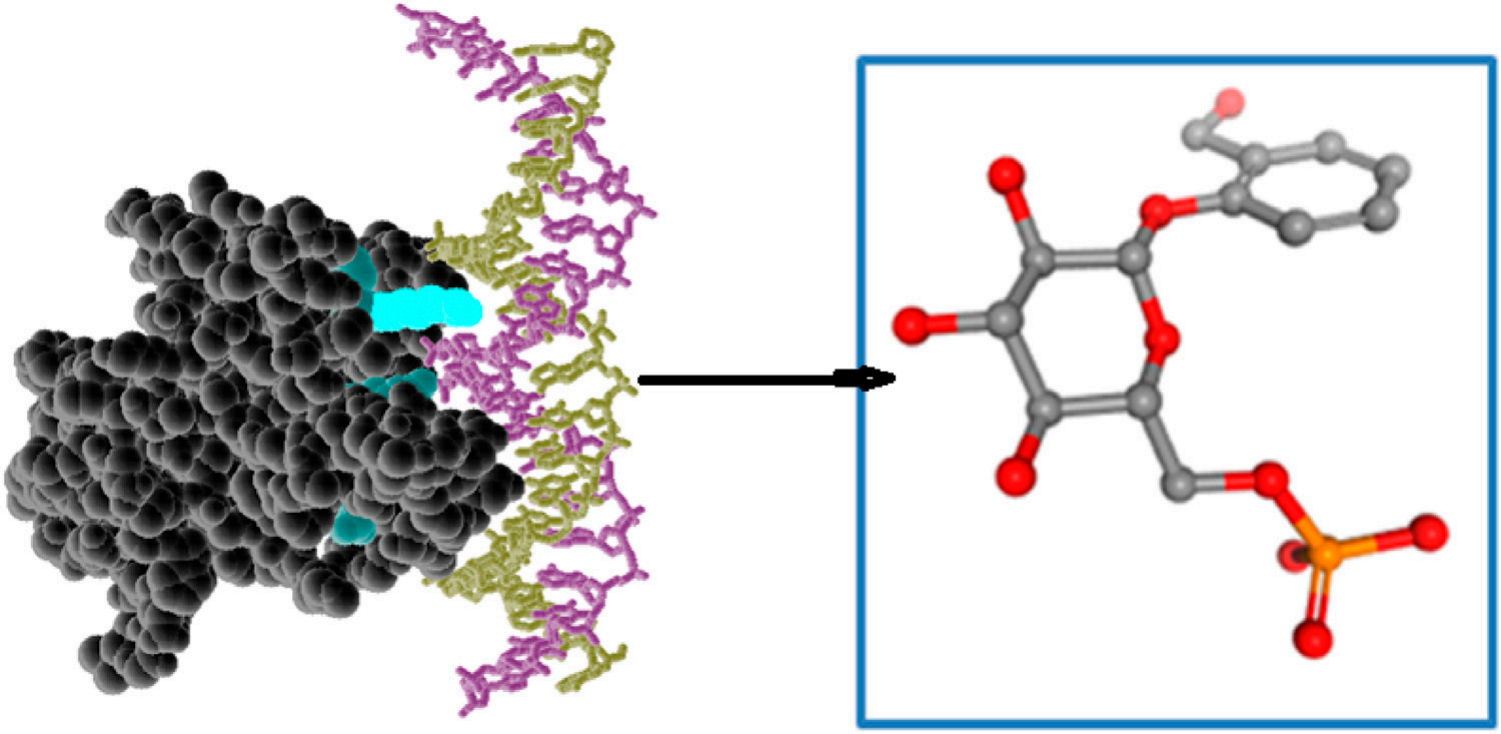
All 3 ligands attached at same binding pockets.

The top three compounds identified by a simulated screening of possible antitumor compounds underwent further investigation based on their drug-likeness, binding preferences, and energies. The top ten compounds’ binding values, ranging from −11.10 to −6.9 kcal/mol, assessed each substance’s ability to attach to the TP53 protein. The best three matches had the lowest binding strengths and most favourable binding energies of all the produced anchored complexes. As seen in Figure 4, these substances were discovered to bind to the same active areas of the TP53 protein. This indicates that these substances might block the protein’s action and might even be turned into effective antitumor medications.

Based on the docking findings, the top three natural compounds (Compound-1, Compound-2, and Compound-3) showed the highest binding energies (−11.10 to −10.3 kcal/mol) in contrast to the other compounds looked at (Table 4). Additionally, the top three compounds interacted with critical areas, suggesting a potential for particular binding to the target protein, P53. We also investigated these compounds’ binding energies and concentrations and their drug-like properties. The compounds with the lowest binding strengths were found to be the most effective at clinging to the active portions of the P53 protein. The selected compounds displayed potential drug-like properties. The P53 protein sequence was also analysed, and the best protein models with the most significant amounts of identity and query coverage were selected for homology-based modelling. Simulation studies of the P53 protein were conducted to identify the top 50 lead matches, and their pharmacophore characteristics were analyzed. We explore that the top 10 lead hits have a pharmacophore fit with particular pockets and loops on the protein’s surface. The findings suggest the possibility of developing these top three natural compounds into influential P53 protein-targeting antitumor drugs.

**FIGURE 5:**
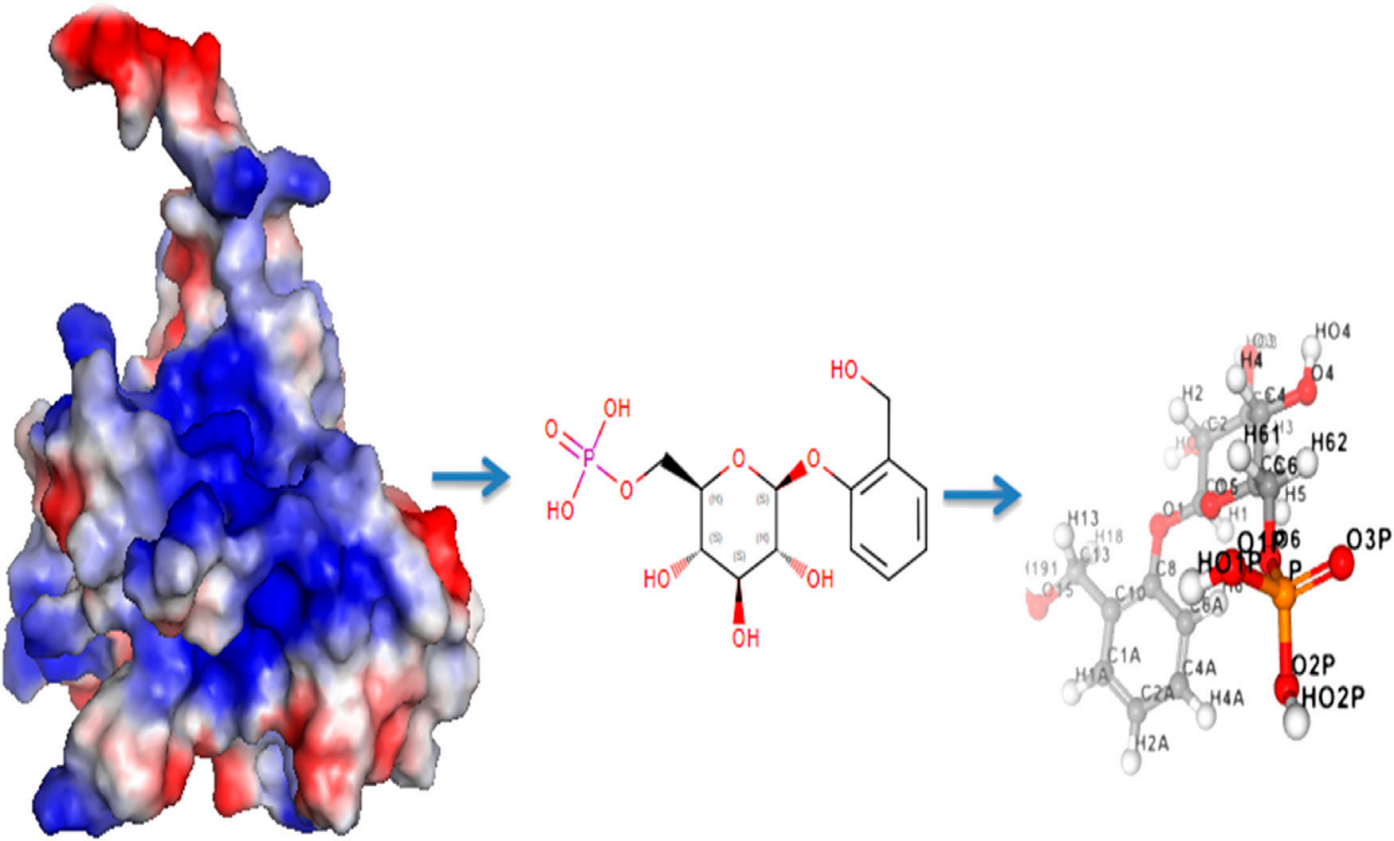
3D docked possess of leading hits.

The binding affinity parameter measures how strongly a ligand—a particle or other small substance—binds to a target protein. The bound sensitivities of ten lead hits against the P53 protein were discovered in this instance. The binding strengths were calculated by considering the drug’s and protein’s intermolecular interactions, including hydrogen bonds, hydrophobic, and electrostatic interactions as shown in Figure 5. The binding energies are expressed in kcal/mol units, with lower numbers indicating stronger binding. With a value of −11.10 kcal/mol, Compound 1 had the highest binding affinity in this instance, suggesting that out of the 10 compounds examined, it attaches to the P53 protein the most firmly. Compound-3 had a slightly lower binding affinity than compound-2, which also had a value of −10.7 kcal/mol, with a value of −10.3 kcal/mol. Compound 4 had the lowest binding affinity of the 10 compounds, with a weight of −8.4 kcal/mol; subsequent compounds had progressively lower binding affinities. More than binding, strengths can affect a drug’s or ligand’s effectiveness; when developing new medicines, thought must also be given to variables like metabolism and toxicity.

The top three natural compounds (Compounds 1, 2, and 3) from the docking analysis of the top 10 lead hits against the P53 protein were discovered to be the most active based on their binding affinities and common chemistry interactions (Table 5). These compounds had the highest binding energies (−10.9 to −9.2 kcal/mol) and bonded to significant sections in the target protein’s active domains. Fewer correlations and weaker binding energies were observed for the other compounds under study. Overall, the results indicate that these top 3 natural ingredients could be used to develop novel cancer therapy drugs. The potent binding sites and common chemical interactions that the bound compounds disclose can be used to create effective inhibitors against the P53 protein.

**TABLE 5:** The binding affinities (kcal/mol) of top-ranked 3 compounds against P53.

Compounds	Binding affinities (kcal/mol)
Compound-1	−10.9
Compound-2	−9.6
Compound-3	−9.2

Using Lipinski’s rule of five, which evaluates the drug-likeness of small compounds, the morphological attributes of the best three hits were examined. The molecules had a molecular weight within the permissible range of 500 g/mol, 10 hydrogen acceptors, 5 hydrogen donors, and 5 LogP values. The LogP number determines the absorption and membrane permeability of the substances. The best three hits’ LogP values varied from 2.06 to 9.02 and were within the permissible absorption range. Additionally, the compounds have a sufficient number of flexible bonds, hydrogen bond acceptors, and donors, which suggests that they have excellent drug action. The best three results’ physical characteristics are shown in Table 6, and it was found that these substances met most of the expected characteristics and lacked cancer potential. According to a study of the ADMET characteristics, the gut can readily take the chemicals. The best three results are good prospects for antitumor medicines based on their physical characteristics and drug-likeness traits.

**TABLE 6:** The analyses of physicochemical Properties of the top 3 selected compounds.

Properties	Compound-1	Compound-2	Compound-3
Molecular weight (g/mol)	420.3877	403.4048	526.7162
Logp (o/w)	2.0649	3.8989	9.0248
H-bond acceptors	6	6	2
H-bonds donors	3	3	2
Rotatable bonds	5	5	1
PSA	74.9200	92.0500	30.2300
Atoms	54	50	91
Rings	5	4	6

The details mentioned in Table 6 provide the physicochemical properties of the top 3 selected compounds, where Compound-1, Compound-2 and Compound-3 molecular weight (g/mol) represents the sum of the atomic weights of all atoms in a molecule, which are composed of 420.3877, 403.4048, and 526.7162 g/mol, respectively. LogP (o/w) is a measure of the solubility of a compound in water (o) compared to octanol (w). The three Compound’s LogP values are 2.0649, 3.8989, and 9.0248, respectively. H-bond acceptors and H-bond donors are related to the hydrogen bonding capacity of a compound with other molecules. Compound-1, Compound-2, and Compound-3 have 6, 6, and 2 H-bond acceptors, and 3, 3, and 2 H-bond donors, respectively. Rotatable bonds indicate the number of bonds that can rotate around their axis within a molecule. Compound-1, Compound-2, and Compound-3 have 5, 5, and 1 rotatable bond, respectively. Polar Surface Area (PSA) measures the size of the polar or charged atoms and groups of atoms in a molecule. Compound-1, Compound-2, and Compound-3 have 74.9200, 92.0500, and 30.2300 PSA values, respectively. The number of atoms and rings in a molecule can influence the properties and interactions of a compound. Compound-1, Compound-2, and Compound-3 have 54, 50, and 91 atoms, and 5, 4, and 6 rings, respectively. The values of these physicochemical properties indicate that the top 3 compounds are drug-like and satisfy Lipinski’s rule of five, which suggests they may have a good chance of becoming a viable drug candidate. The ADMET analysis indicates that these compounds are non-carcinogenic and can be easily absorbed by the intestine.

### 3.9 MD simulation

**FIGURE 6:**
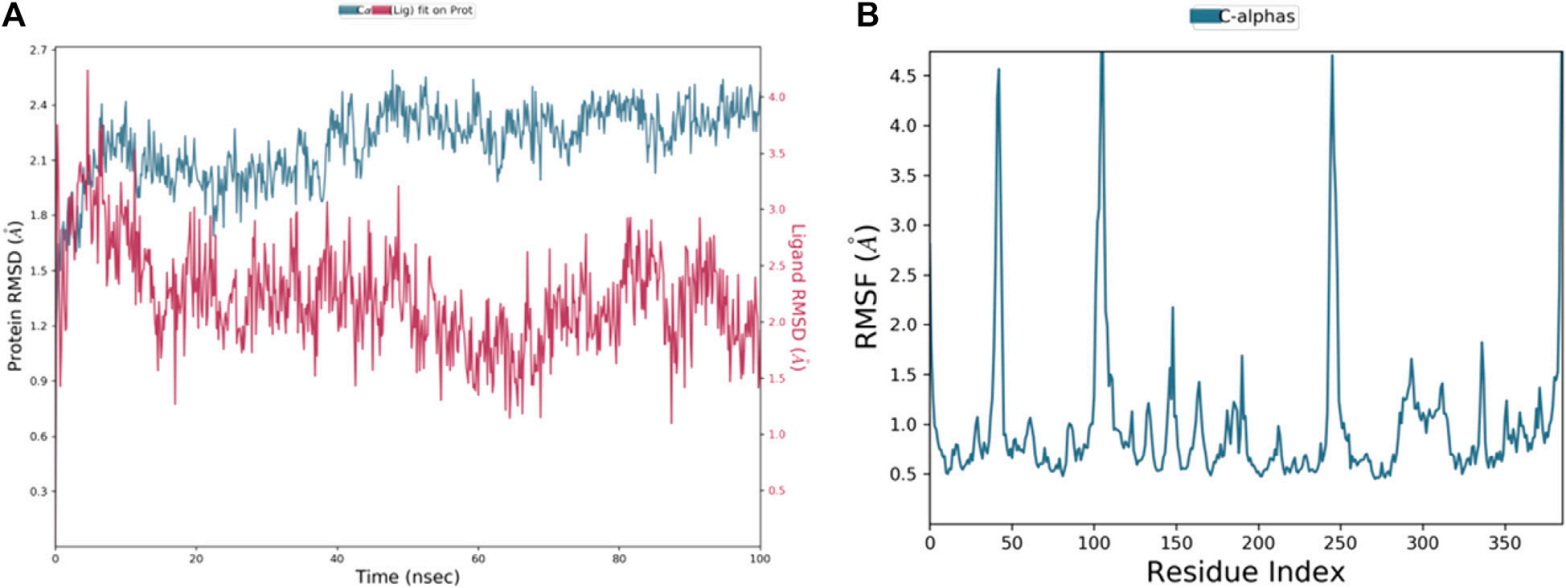
RMSD and RMSF plots for a ligand-protein complex during 100 ns of MD simulation. **(A)** Representation of ligand–protein complex. **(B)** Major fluctuations at peak 1, 2, 3, and 4.

Molecular dynamics (MD) simulations are an effective tool for investigating the dynamic behavior of proteins at biologically relevant timescales (Rather et al., 2020). In this study, MD simulations were used to examine how the presence of ligands affects the stability and conformational changes of the TP53 protein. The RMSD plot is a widely used method for evaluating the structural stability of proteins during MD simulations, which measures the deviation of the protein backbone conformation from the starting structure as a function of time. According to the results of this study, the RMSD plot for the TP53 protein displayed an initial increase in the C-α backbone from 1 ns to 25 ns, indicating significant structural rearrangement. This was followed by a gradual decrease in RMSD from 26th ns to 30th ns, indicating that the protein was becoming more stable. Peaks in the RMSD values from 35 ns to 40 ns suggested that the protein was undergoing further conformational changes. The gradual decrease in RMSD from 65 ns to 100 ns suggested that the protein had finally achieved a stable conformationas shown in Figure 6.

As shown in Figure 6A, the ligand’s RMSD variations were congruent with those of the protein C-backbone, proving that the ligand was stablely attached to the protein throughout the experiment. The bound complex’s average RMSD over the course of the 100 ns exercise was 3.2 Å, showing that the protein-ligand complex was largely steady. Because the bound complex’s average RMSF was 4 Å, some structural changes may have occurred in the protein during the experiment. Another popular technique for examining the kinetics of proteins in MD models is the RMSF image. Each protein residue’s departure from its typical location is tracked during the exercise. In this research, the TP53 protein’s RMSF histogram for positions 1–95 revealed a rise, with a highest value of 5 Å.This indicates that these residues were more malleable during the exercise and experienced more structural changes. Other positions showed modest variations with an average RMSF of 3 Å. Residues with a value of 4.8 showed an increase in peaks 3 and 4 Å, suggesting that these residues were experiencing substantial structural changes. Altogether, the outcomes of the MD models indicate that the chosen substances can attach to TP53 and maintain its shape, thereby preventing the development of cancer cells. The protein-ligand complex’s stability was examined using a number of metrics, such as the RMSD, RMSF, and ligand characteristics, and it was found to be a stable, closely bonded structure with a high degree of density. These discoveries shed light on the protein-ligand interaction process and may help create new cancer treatments.

The departure of the shape of the ligand-protein complex from the initial structure over time is depicted by the RMSD (Root Mean Square Deviation) diagram for a ligand-protein complex during a 100 ns Molecular Dynamics (MD) simulation. The RMSD, which is used to track the stability and progress of the simulation, is a measurement of the average distance between the atoms of the simulation structure and the reference structure (typically the beginning structure). The RMSD figure frequently exhibits an early rise, a peak, and a steady rise over time. While the peak shows the stability of the complex throughout the exercise, the initial rise results from the system relaxing from its original structure. The steady rise over time results from the system’s tiny variations adding up, which can have a significant systemic impact.

The main peaks and valleys of the ligand-protein combination during the experiment are depicted in the RMSF (Root Mean Square Fluctuation) Figure 6B. The complex’s most malleable areas or those undergoing the greatest structural changes during the exercise are indicated by the peaks in the RMSF image. The average departure of each residue’s location from its normal position throughout the experiment is represented by the RMSF, which is computed for each residue in the protein or ligand. The complex’s area of high flexibility or movement is indicated by peak 1 Å in the RMSF image, while the region of moderate flexibility is demonstrated by peak 2 Å, Peaks 3 Å, and 4 Å, could be localised structurally changing areas that interact with fluid molecules or other regions of the complex. The analysis of these peaks can reveal details about the dynamics of the complex and point out areas crucial to its stability or functionality.

The root mean square distance of all parts from the protein’s centre of mass is known as the radius of gyration (Rg), which gauges how dense a protein’s structure is. It shows how crowded and how much room the protein’s auxiliary components fill. Proteins with bigger Rgs have structures that are longer or more open, whereas proteins with smaller Rgs have structures that are closer together. A closely bonded complex in the context of protein-ligand binding denotes intimate interaction between the protein and the ligand, resulting in a lower total Rg of the complex than the isolated protein or ligand. This could mean a high propensity for the protein and receptor to bind. The stability and kinetics of protein-ligand interactions are frequently assessed using MD models and the RMSD (Root Mean Square Deviation) and RMSF (Root Mean Square Fluctuation) studies. The RMSF measures the average variation in each molecule or peptide throughout the experiment, while the RMSD determines how much the protein-ligand combination deviates from its original structure. The protein-ligand complex is physically stable, and the protein and ligand are closely attached if the RMSD/RMSF of the protein-ligand complex stays constant throughout the exercise. On the other hand, if the complex is experiencing structural alterations or the protein-ligand association is feeble, the RMSD/RMSF ratios are significant.

We analyze the phytochemicals based on the absence of significant structural changes in the RMSD/RMSF plot during the simulation. This indicates that the compounds are structurally stable and not undergoing any major conformational changes, per earlier researchers (Hafiz Muhammad et al., 2022). This is a significant result as it suggests that the phytochemicals aren’t degrading or becoming unstable during the simulation. Therefore, the observed interactions with the protein likely represent their actual behaviour *in vivo*. The study’s results suggest that the protein-ligand complex is tightly bound, structurally stable, and compact, indicating a strong binding affinity between the protein and ligand. These findings are consistent with previous research and provide insights into the structure-function relationships of the complex.

### 3.10 Protein structure prediction analyses

**FIGURE 7:**
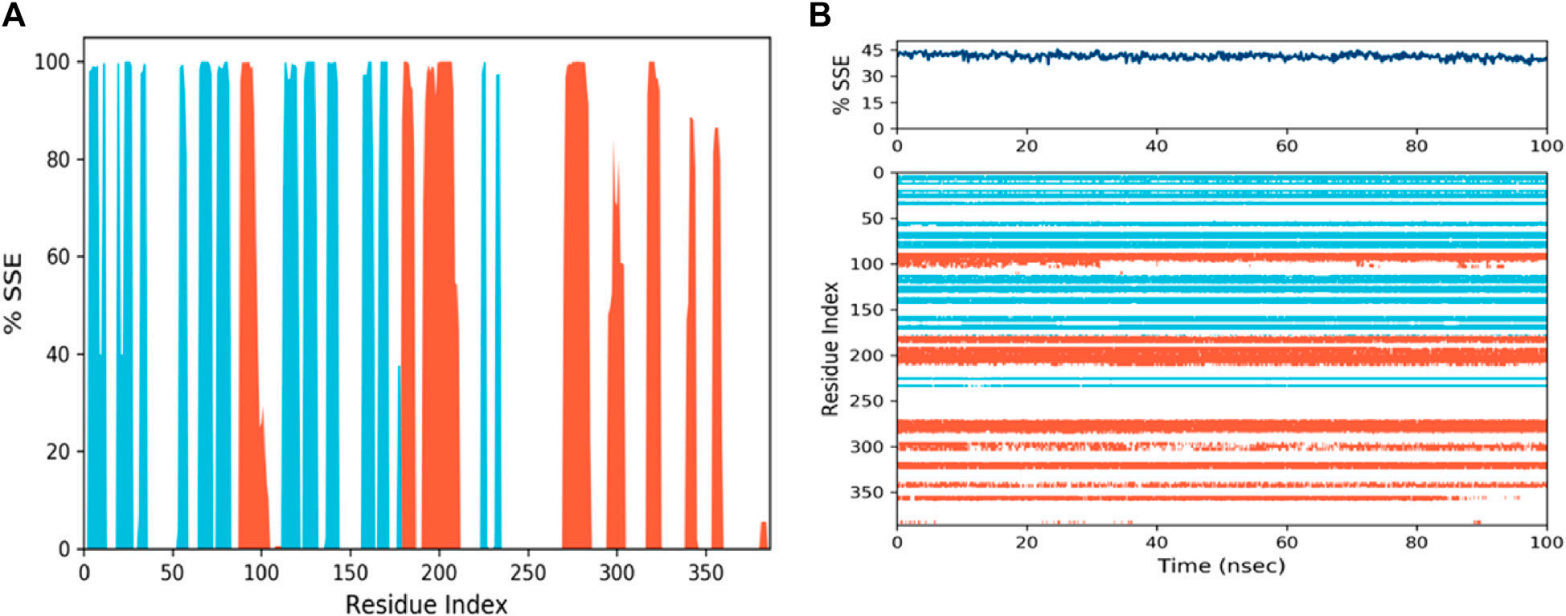
Visualization of the secondary structures of alpha helices, beta-strands, and other secondary structures in protein structure. **(A)** SSE distribution by residue index: red peaks = helices, blue peaks = beta-strands. **(B)** SSE composition for each trajectory frame and assignment for each residue over time.

The study of secondary structure elements (SSE) offers details about the protein’s molecular alterations during the MD simulation. As you pointed out, the findings in Figure 7 shows that the protein’s helices and strands experienced a major shape shift, which raises the possibility that the protein endured a substantial structural reorganisation. The protein was highly dynamic and many areas experienced significant structural changes, as evidenced by the fact that 43.78% of the protein’s SSE were impacted during the 100 ns simulation. The study of several ligand about radius of gyration, intramolecular hydrogen bonds, molecular surface area, solvent-accessible surface area, and polar surface area, showed ligand is firmly attached to the protein despite these structural alterations (PSA). The fact that these characteristics barely changed suggests that the ligand was firmly attached to the protein and that its interactions with the protein were very steady. According to these results, the protein-ligand complex is highly stable and closely bonded even though the protein underwent substantial structural changes during the MD simulation. This aligns with earlier research’s findings and emphasises the importance of comprehending the kinetics and stability of protein-ligand interactions to create more successful drug design methods. Overall, data offers insightful information about the stability and structural changes of a protein-ligand complex during an MD simulation, and these discoveries can be used to guide future studies into the creation of new medicines.

The secondary structure components of the protein are represented visually in the protein structure by the SSE distribution by residue index (Figure 7A). In the diagram, alpha helices are represented by the crimson peaks and beta strands by the blue peaks. Plotting the spread of these components along the protein chain enables a rapid evaluation of the protein’s general secondary structure. The SSE makeup for each motion frame throughout the experiment is shown in the figure below (Figure 7B). This diagram displays the spread of beta strands, alpha helices, and other secondary structures at various modelling time points. It makes it possible to see how the SSE makeup varies over time and can be helpful in pinpointing instances of instability or structural change. The image at Figure 7 bottom tracks each residue’s SSE designation over time. This image thoroughly examines how each residue’s SSE evolves throughout the exercise. The image can help find the stability of particular secondary structures or locate specific acids that may be engaged in structural shifts. Overall, the SSE representation offers essential knowledge about the secondary structure components of the protein and how they evolve throughout the MD simulation. Researchers can learn more about the protein’s stability and structural changes that may be crucial for ligand binding and function by examining changes in the SSE makeup.

**FIGURE 8:**
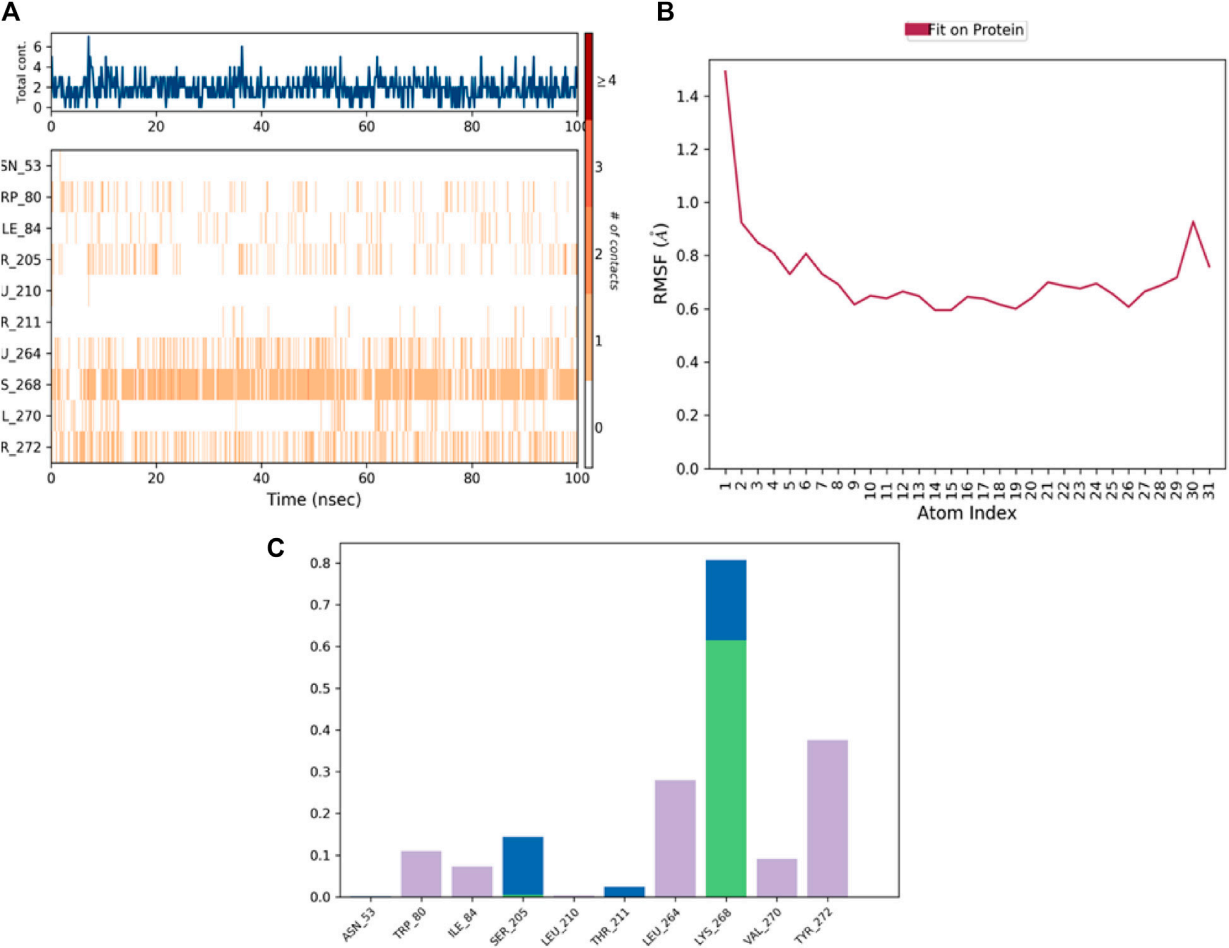
A schematic of ligand-ligand interactions. **(A)** Protein-ligand contacts categorized by interaction. **(B)** Timeline of H-bond and hydrophobic contacts between protein and ligand. **(C)** Schematic diagram of ligand atom interactions with protein residues.

The information regarding the ligand components involved in interactions and their interaction with protein residues, specifically amino acids, is depicted in (Figure 8A). This information, which illuminates the critical relationships between the ligand and protein, can help develop novel ligands or improve already existing ones. The ligand features graphic shows the ligand’s radius of gyration (rGyr), molecular surface area (MolSA), solvent accessible surface area (SASA), and polar surface area (PSA) as shown in (Figure 8B). These traits can provide details regarding the ligand’s stability and protein interactions. A safe ligand, for instance, should have a minimum radius of gyration to form intramolecular hydrogen bonds. The ligand’s molecular surface area can be used to estimate its size. Its polar surface area and liquid accessible surface area can be used to determine its solubility and polarity. Figure 8C shows the interactions in protein-ligand junctions and categorises them by category. They illustrate the ligand’s interactions with specific protein areas containing amino acids. Among the different types of interactions that can occur are electrostatic interactions, hydrophobic contacts, and hydrogen bonds. The hue labelling in the picture represents the number of distinct connections of a specific peptide with the ligand. Darker orange indicates that a peptide has been exposed to the receptor more than once. The links between the ligand and the protein can be improved by identifying key amino acid sequences implicated in ligand binding.

## 4 Conclusion

The study investigated the presence of phytochemicals and antioxidant properties in the Amomum subulatum seeds. W*e* confirmed the presence of critical phytochemical groups (alkaloids, tannin, saponin, phlobatinin, and cardic glycoside) responsible for the plant’s medicinal properties. The *A. subulatum* plant’s ability to scavenge reactive oxygen species (ROS) makes it a potential candidate for therapeutic use, particularly in treating cancer. In this study, our results revealed that the extract comprised 9.4% ± 0.04% alkaloid and 1.9% ± 0.05% saponin. We also performed DPPH analysis and found that the methanol extract (79.82%), BHT (81.73%), and n-hexane extract (51.31%) exhibited considerable antioxidant activity.

Furthermore, we assessed the extract’s ability to inhibit oxidation and observed that Methanol (83.42%) and BHT (90.25%) had the most significant inhibitory effects. We found that Compound-1 had the best pharmacophore match value (53.92), with others ranging from 50.75 to 53.92, where docking results showed that the top three natural compounds had the highest binding energies (−11.10 to −10.3 kcal/mol) and bound to significant regions in the target protein’s active domains. The Molecular Dynamics (MD) simulations indicated that the ligand was tied to the protein with substantial conformational changes in the protein structure. The study also identified three compounds that exhibit promising effects against cancer cells, with marked effects on the TP53 protein and the P53 pathway.

The computational models provide insight into protein-ligand interactions and binding affinities. They may serve as a model for developing innovative, less toxic, and highly effective drugs for cancer treatment. The study’s findings highlight the potential of natural compounds for the development of novel cancer therapies.

## Data Availability

The original contributions presented in the study are included in the article/supplementary material, further inquiries can be directed to the corresponding authors.
